# FIJI Macro 3D ART VeSElecT: 3D Automated Reconstruction Tool for Vesicle Structures of Electron Tomograms

**DOI:** 10.1371/journal.pcbi.1005317

**Published:** 2017-01-05

**Authors:** Kristin Verena Kaltdorf, Katja Schulze, Frederik Helmprobst, Philip Kollmannsberger, Thomas Dandekar, Christian Stigloher

**Affiliations:** 1 Division of Electron Microscopy, Biocenter, University of Wuerzburg, Wuerzburg, Germany; 2 Department of Bioinformatics, Biocenter, University of Wuerzburg, Wuerzburg, Germany; 3 Molecular Biotechnology and Functional Genomics, Technical University of Applied Sciences Wildau, Wildau, Germany; 4 Center for Computational and Theoretical Biology, University of Wuerzburg, Wuerzburg, Germany; Northwestern University, UNITED STATES

## Abstract

Automatic image reconstruction is critical to cope with steadily increasing data from advanced microscopy. We describe here the Fiji macro 3D ART VeSElecT which we developed to study synaptic vesicles in electron tomograms. We apply this tool to quantify vesicle properties (i) in embryonic *Danio rerio* 4 and 8 days past fertilization (dpf) and (ii) to compare *Caenorhabditis elegans* N2 neuromuscular junctions (NMJ) wild-type and its septin mutant (*unc-59(e261)*). We demonstrate development-specific and mutant-specific changes in synaptic vesicle pools in both models. We confirm the functionality of our macro by applying our 3D ART VeSElecT on zebrafish NMJ showing smaller vesicles in 8 dpf embryos then 4 dpf, which was validated by manual reconstruction of the vesicle pool. Furthermore, we analyze the impact of *C*. *elegans* septin mutant *unc-59(e261)* on vesicle pool formation and vesicle size. Automated vesicle registration and characterization was implemented in Fiji as two macros (registration and measurement). This flexible arrangement allows in particular reducing false positives by an optional manual revision step. Preprocessing and contrast enhancement work on image-stacks of 1nm/pixel in x and y direction. Semi-automated cell selection was integrated. 3D ART VeSElecT removes interfering components, detects vesicles by 3D segmentation and calculates vesicle volume and diameter (spherical approximation, inner/outer diameter). Results are collected in color using the RoiManager plugin including the possibility of manual removal of non-matching confounder vesicles. Detailed evaluation considered performance (detected vesicles) and specificity (true vesicles) as well as precision and recall. We furthermore show gain in segmentation and morphological filtering compared to learning based methods and a large time gain compared to manual segmentation. 3D ART VeSElecT shows small error rates and its speed gain can be up to 68 times faster in comparison to manual annotation. Both automatic and semi-automatic modes are explained including a tutorial.

This is a *PLOS Computational Biology* Software paper.

## Introduction

The accurate interconnection and signal transmission of chemical synapses is crucial for the processing of stimuli in the brain, which is incisive for all mental processes, ranging from simple reflexes to control of complex behavioral traits and learning. Furthermore, the precise chemical signal transduction in the peripheral nervous system through neuromuscular junctions is essential for precise coordination of the locomotor system. Studying the ultrastructural architecture of chemical synapses is central to the understanding of synaptic function. Due to the small size of synaptic components such as synaptic vesicles far below the resolution limit of diffraction limited light microscopy, high resolution techniques studying the ultrastructure of synapses are essential in order to acquire a broad understanding of important neurological functions like exocytosis and synaptic recycling. Transmission Electron Microscopy (TEM) is the standard technique that provides high resolution images of cells and tissues down to the nm-scale in the lateral x- and y-dimensions. The main limit of TEM to study synaptic architecture is the limited resolution in the z-dimension, e.g., its section thickness typically ranging between 40 and 100 nm for ultra-thin sections. Electron tomography is an advancement of TEM to circumvent this limitation [1]. The most important difference in tomography is the variable viewing angle in which electron microscopic pictures of an object are recorded. By combining the information of all images of one tilt-series, a virtual section series can be reconstructed *in silico* that has a near isotropic resolution of typically 4 to 5 nm in x, y and z which was determined by measuring the “unit membrane” thickness [2–6]. The production of tomograms and the following combination and computation of the final tomogram is a time-consuming process. However, for further detailed analysis and generation of data an even more time consuming annotation of the structure of interest is necessary. We used electron tomography for fine architectural analysis of synaptic vesicle pools of neuromuscular junctions (NMJs), synapses that innervate muscles. This way we compared NMJs of different developmental states and mutants with morphological alterations to wild types. Up to now, we used the software package IMOD for manual reconstruction [7]. Hence, every vesicle had to be manually annotated by scrolling through the virtual slides in order to estimate the vesicles’ maximum diameter. In the next step the center of the vesicle was determined. Finally, the size of the annotated vesicle was adjusted to the size of the vesicle in the virtual slide representing the maximal diameter, the final size of which may vary depending on the executing person. Thus manual annotation is subjective and definitely lacks precision. For extensive studies, the elaborate manual annotation step is very time-consuming and therefore can be considered as the limiting factor of tomography analysis. Regarding tomographic procedures used for medical diagnosis, e.g., computed tomography (CT) and magnetic resonance imaging (MRI), various algorithms and standardized software for automated image analysis already exist [8]. In contrast, for annotation of specific biological structures such as synaptic vesicles in electron tomograms, user friendly, freely available and ready to apply tools are often lacking, as they have to be labor-intensively specifically developed and adapted for the biological problem, for instance regarding plankton detection [9].

The majority of segmentation algorithms for electron microscopy works on 2D slices from anisotropic imaging methods such as serial section microscopy, and does not utilize the full 3D information from tomographic stacks. Only a small number of approaches were developed specially for isotropic 3D data, specifically to annotate mitochondria [10], membranes [11], filamentous structures [12] and other macromolecular structures [13], [14], reviewed in [15], [16]. Recently, the field of connectomics has sparked active research in machine-learning based methods to segment neurons and their synapses from 3D electron microscopy data [17–19]. Typically, these methods require expert-level knowledge to be implemented and applied, and are not widely available as a ready-to-use tool. In the case of trainable algorithms, e.g., *ilastik* [20], time-consuming manual labeling of training data is required (comparison of 3D ART VeSElecT to ilastik, see supplemental material, S1 Fig). These factors have so far limited the adoption of most published methods for automated 3D EM image annotation by a wider community. As a result, the annotation of biological structures, and specifically of vesicles, in electron tomography data is to date still being carried out mostly manually.

The goal of this project was to overcome this limitation by implementing an automated method for segmentation and quantification of synaptic vesicles in electron tomograms that can be used by non-experts and is purely algorithmic, i.e., does not require manually labeled ground truth data. We aimed for easy and wide applicability of our tool to ensure systematic, reproducible and consistent quantification across different laboratories. For this reason we developed two macros for the open source image processing software Fiji [21], which are able to automatically find and annotate vesicle structures in electron tomograms. The first macro, the recognition macro, automatically annotates vesicles. The second one depends on the reconstruction macro and is used to extract information, like vesicle diameters, vesicle volume and vesicle distances to the closest neighbor vesicles, after the annotation step. The development of such software is a challenging task, as electron tomography provides noisy low contrast images, therefore even simple structures can be difficult to identify not only for humans but even more for computers. Thus, our macro uses different filters for smoothening, contrast enhancement and accentuation of edges. Note that automated vesicle registration and characterization was implemented in Fiji as two macros (registration and measurement). This flexible arrangement allows in particular reducing false positives by an optional manual revision step whereas all other steps run all fully automatically.

To test the macro for its functionality and show potential fields of application, we used it for the analysis of two different issues. First we compared embryonic zebrafish NMJs of different developmental stages. Therefore we applied the macro on datasets of embryonic zebrafish NMJs 4 days post fertilization (dpf) and 8 dpf which have already been annotated and analyzed manually [22]. We compared the manually annotated data to the output of our automated framework in order to ascertain its efficiency. Additionally, we applied the macro on *C*. *elegans* N2 NMJs and its septin mutant (*unc-59(e261)*) to compare mutants and control organisms for morphological differences to ensure the macros functionality on other organisms and in various fields of application.

Septins are highly conserved cytoskeletal proteins that play a role in cell division and compartmentalization and are functionally attributed to important neuronal processes like: neuronal development, axon guidance, axonal transport, vesicular trafficking and exocytosis [23–25]. Septins are important factors in many neurodegenerative diseases [26], [27]. Mutations affecting these proteins have strong influence on human health. Septins are found in most eukaryotes and occur in a high number of various isoforms. Humans for example have 13 septin genes [28], the yeast *Saccharomyces cerevisiae*, has seven different septins [29–31]. However, *C*. *elegans* has only two different septin genes, *unc-59* and *unc-61*, which enormously facilitates the examination of basic septin function and thus makes it the perfect model organism to further investigate their function. UNC-59 and UNC-61 are named after the phenotype of loss-of-function mutants, that are characterized by their uncoordinated movement [32]. The uncoordinated behavior was previously linked to cytokinesis defects in postembryonic neuroblasts, which was disproved by demonstrating that uncoordinated movement also occurs in the absence of cytokinetic failure[33–36]. Thus axonal migration defects have been supposed as reason for uncoordinated behavior [33]. Still the ultimate reason for the “unc” phenotype remains to be discovered. Therefore, we applied our macro to compare quantitative features of synaptic architecture of N2 versus *unc-59(e261)* mutant NMJs.

## Results

For a better understanding of our terminology we shortly describe our definitions for “automated” and “semi-automated”. The macro itself automatically identifies and reconstructs vesicles without manual intervention. The only manual tasks are the input of parameters (definition of search results) at the beginning and the semi-automated area selection. The manual editing step is independent and optional and improves the result, but is not part of the tool itself. The tool however facilitates this manual post-processing step. This is why the registration of the vesicles is performed automatically by the macro and only the complete workflow including manual proof-reading results in a semi-automatic 3D reconstruction.

### Algorithms of 3D ART VeSElecT: Automated registration and characterization

**Fig 1:**
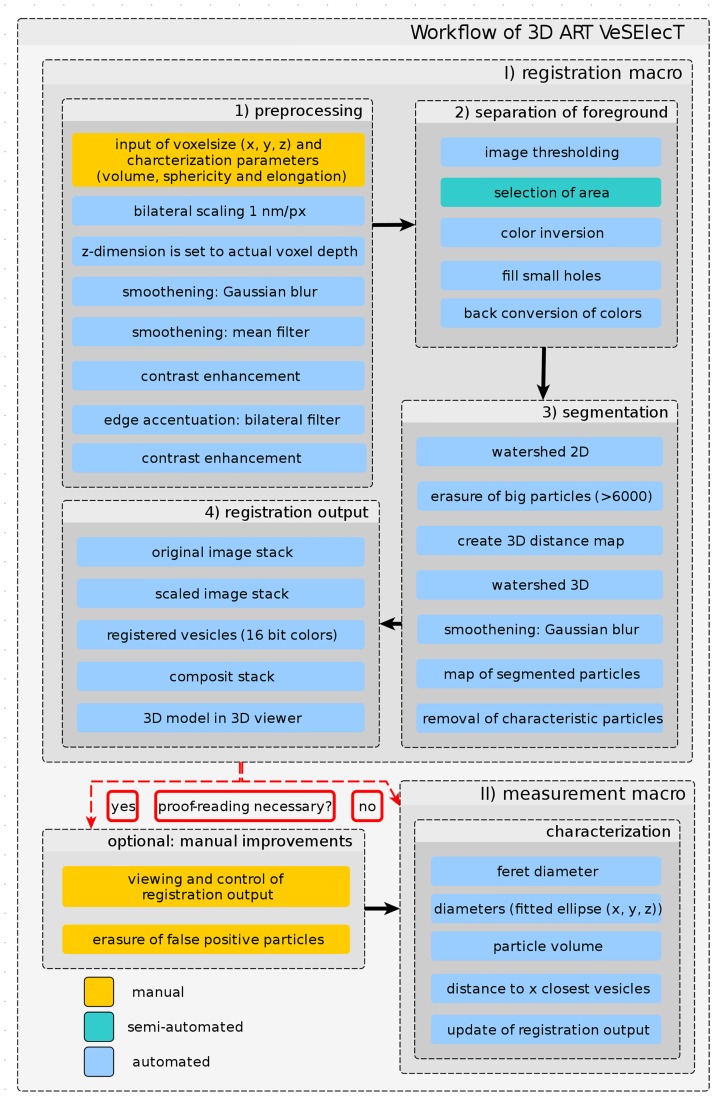
Workflow of vesicle annotation using 3D ART VeSElecT. First, the automated registration macro is used, which scales the tomogram via user input of the pixel size and applies various filters in the preprocessing step. Afterwards the foreground is separated, the user semi-automatically selects an area of interest, and the macro applies the watershed algorithm for vesicle segmentation and registration. Second, an optional manual proof-reading step can be applied here, if necessary. Finally, the automatic measurement macro is used to extract results using certain characteristics. All manual steps are colored in yellow, semi-automated steps are in turquoise, automated steps are in blue.

The automated registration and characterization of the synaptic vesicles was implemented as two macros for Fiji [21]. Fiji is a distribution of ImageJ which includes a wide range of plugins for biological image analysis. The first macro allows the segmentation of vesicles and includes steps for the preprocessing, separation of the foreground, removal of interfering components and segmentation of individual vesicles (Fig 1: (I) registration macro). Afterwards all registered vesicles can be characterized with the use of the second macro (Fig 1: (II) measurement macro). This separation allowed a better exclusion of falsely registered particles from the final characterization. All steps of the analysis are explained in detail below.

#### Preprocessing

To allow a reproducible routine for different image resolutions and the determination of real vesicle sizes the image-stack was scaled to 1 nm/pixel in x- and y-direction using bilateral scaling. For the z-dimension, no rescaling was applied and the distance between the stack images was set to the actual voxel depth, in order to maintain the proper spatial dimensions without generating additional interpolated images. The pixel per nm resolution for the image-stack was set over user input. Afterwards the contrast was enhanced via histogram stretching over the entire intensity range, whereas 0.4% of the pixels were allowed to become saturated. The stack was then smoothed with a mean filter (radius = 3.45 px) to lower the image noise. Additionally, a bilateral filter was applied for noise-reduction and edge-preservation.

#### Separation of the foreground

The separation of foreground and background was performed for every stack image individually via image thresholding based on the mean brightness.

To reduce the influence of interfering components within the images, a semi-automated cell selection was integrated. For this the synapse wall has to be traced by hand in the first and last image of the stack. Based on these selections the regions of interest (ROIs) for the remaining images in the stack were interpolated and image parts outside of these selections are excluded from further analysis.

Since the inside of the vesicles often had similar intensity values as the background, holes within the vesicles were frequently generated. These were filled in an additional custom processing step: Due to the tight arrangement of all components, a limitation of the hole size was integrated, otherwise other parts of the background would have been filled, resulting in an incorrect separation. For this, the image-stack was dilated to fill small defects that interfered with the following steps. Afterward the image-stack was brightness-inverted to register all holes with a size between 0–575 pixels. All registered holes were then filled and the stack was inverted back to the original image resulting in a stack with correctly separated background and foreground.

#### Removal of interfering components and segmentation of individual vesicles

Since various components are contained with a high density within the cells, almost all cell parts were interconnected in the thresholded stacks. In order to extract the round vesicles, a watershed segmentation was performed in a two-step process: first a 2d segmentation was done, then a second 3D segmentation was performed using methods from the 3D ImageJ Suite [37]. This two-step approach helped to reduce the computational effort and memory usage considerably. Additionally it allowed a more precise elimination of interfering components, which was done between both segmentation steps via an erosion of the stack-images and the removal of particles with a size above 6000 pixel.

For the 3D segmentation, a 3D Distance map was created and smoothed with a Gaussian blur of 3.45 in order to remove artifacts on the particle edges. Based on the created distance map, a 3D watershed segmentation was performed (image threshold = 2 & seed radius = 2.3 px) which resulted in a map of separated particles with different colors. In a final step all particles were registered with the 3D RoiManager plugin and particles which did not match predefined characteristics (volume, sphericity and ellipse fitting; characteristics are depending on the organism: see Materials and Methods) were automatically removed.

#### Registration output

As output of the registration steps the following windows are displayed:

- The original image-stack
- The scaled image-stack
- A map with the registered vesicles in 16-bit colors
- A composite image-stack, where the registered particles are superimposed into the original stack
- A 3D representation in the 3D Viewer plugin [38]

All vesicles are registered within the 3D RoiManager plugin and can thus be selected individually from within the stacks. The name of the ROIs corresponds to the color of the respective particle in the 16-bit color map. This allowed the manual exclusion of potentially falsely registered particles.

#### Characterization

In the second macro, various characteristics are determined for every registered vesicle based on the Measure 3D function of the 3D RoiManager. This includes the Feret diameter, diameters in x, y and z-direction based on a fitted ellipse (ordered after size and not orientation), particle volume and the distance to the n closest vesicles, where n was set in the beginning of the macro. All measurements are given in nm. Additionally all pictures are updated to show only vesicles that are included in the analysis.

### Comparison of manual and automated annotations exemplified on 4dpf and 8dpf embryonic *Danio rerio* neuromuscular junctions (NMJ)

In order to test 3D ART VeSElecT for its functionality and deliver a proof of principle for our software illustrating its performance, we compared the results generated by the macro to data which have already been analyzed manually. Therefore we used tomograms of NMJs of zebrafish embryos (4dpf and 8dpf). In a previous publication of Helmprobst et al. [22], systematical differences were detected between the different developmental states of embryos. We used our macro to analyze this issue a second time, therefore applying 3D ART VeSElecT for comparison of manual and automated results and to check whether the differences between embryos of different developmental states can be confirmed by our new analysis tool.

**Fig 2:**
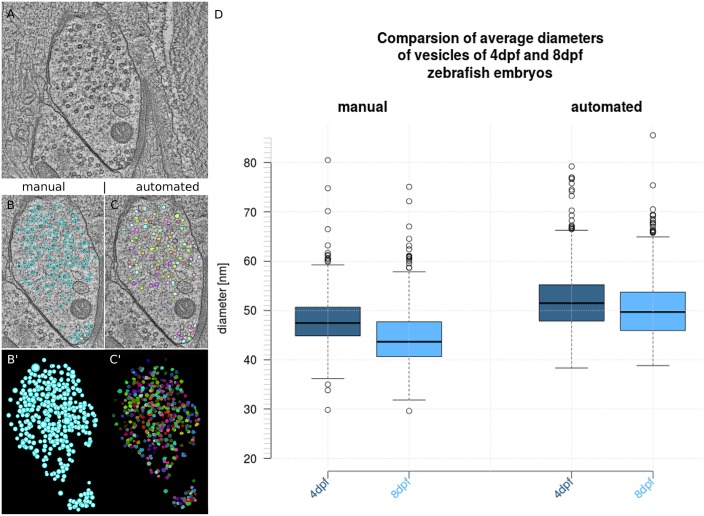
Analysis of embryonic zebrafish NMJ using 3D ART VeSElecT in comparison to manual analysis using IMOD. We show in Fig 2 A) the original tomogram of 4dpf zebrafish NMJ, in Fig 2 B) the manual reconstruction is included in the tomogram of A), in B') the 3D reconstruction of the manual annotation (vesicles are colored in light blue) is shown. This is compared to Fig 2 C) which shows the semi-automated vesicle recognition overlaid with the original tomogram, and C') which shows the vesicle pool of the semi-automated annotation as 3D reconstruction (vesicles are in arbitrary colors). In D) boxplots show the results of the comparison of 4dpf and 8dpf zebrafish embryos using manual annotation (left) and semi-automated annotation (right). The box of the box plots shows the mid-50% of data. The line in the box represents the median of all data. Whiskers end at lowest value within 1.5 interquartile range (IQR) of the lower quartile and at the highest value within 1.5 IQR of the upper quartile. Data that is not included in between both whiskers are plotted as outliers with a dot.

Fig 2A shows a tomogram of a 4 dpf zebrafish embryo neuromuscular junction (NMJ). The NMJ was reconstructed in two different ways, first, manually using the IMOD software package [39] (Fig 2B and 2B’) and second, using the developed 3D ART VeSElecT macros for Fiji, including additional manual proof-reading [21] (Fig 2C and 2C’). The visual comparison shows, that vesicles are very accurately and efficiently recognized by the macro. The distribution and position of the annotated vesicles of the automated reconstruction are readily comparable to the manual annotation of the synapse. Furthermore, closer examination shows that individual vesicles can be recognized and identified in both reconstructions.

**Fig 3:**
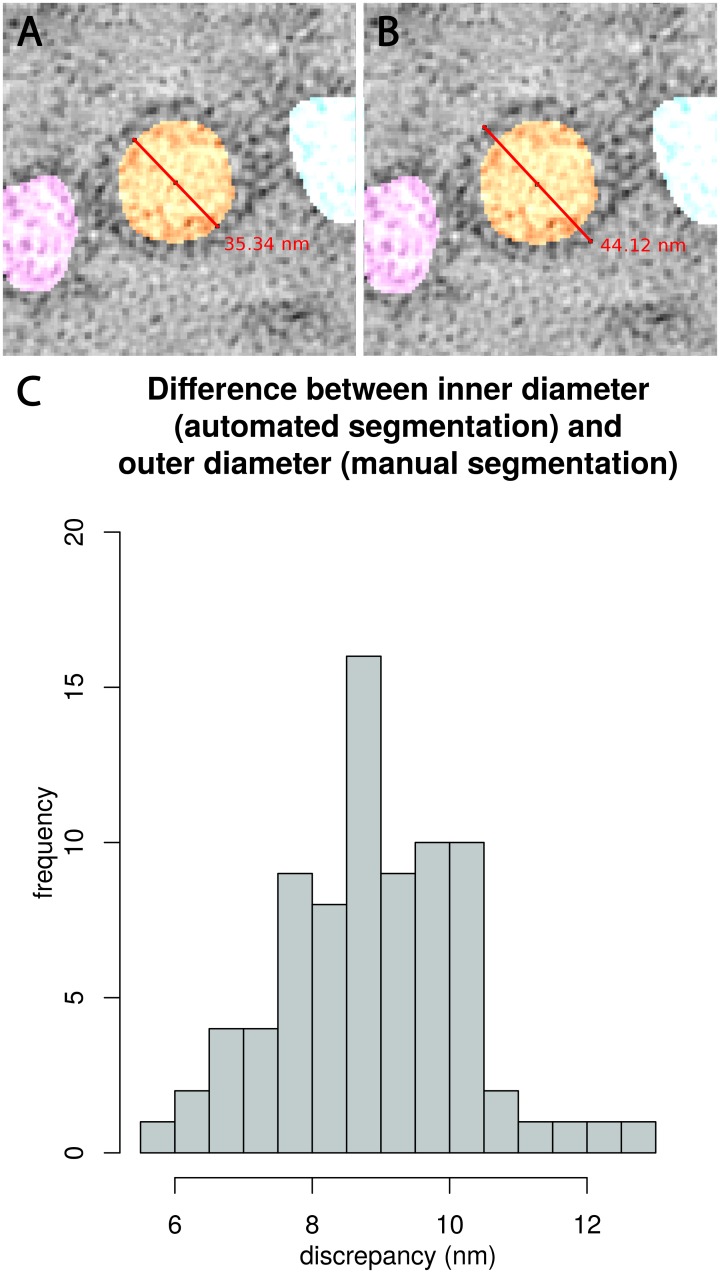
Measurement of inner and outer synaptic vesicle diameter. In order to get an approximate value of the discrepancies between vesicle diameters of manual and automated measurement we applied the Fiji measurement tool. Fig 3 (A) shows the inner diameter of a vesicle that was annotated by 3D ART VeSElecT, (B) shows the outer diameter. Fig 3 (C) gives the results of the discrepancy of inner and outer diameter of all measured vesicles shown as a histogram (number of measurements = 80).

Manual measurement and automated measurement are two different approaches to get average vesicle diameters. For manual measurement as published in Helmprobst et al. [22] the software IMOD [39] was used to place a sphere into every vesicle, the size was adjusted in a way that it includes the vesicle membrane as shown in Fig 2B. Radius and diameter are extracted from this sphere. The mean of the manual measurement is 47.86 ± 4.98 nm for 4dpf and 44.45 ± 5.49 nm for 8dpf embryos which results in a discrepancy of 3.41 nm (Fig 2D) between 4dpf and 8dpf embryos. In contrast to the manual reconstruction method, the macro registers automatically the interior vesicle lumen without the vesicle membrane (Fig 2C and 2A). To visualize the results of the semi-automated reconstruction, an average diameter on basis of diameter 1, 2 and 3 was calculated, to get an average size for each vesicle. Diameters from manual and automated measurement differ systematically, in a way that automatically generated results are always smaller than manual measurements. This can be explained by the fact, that both measurements have slightly divergent approaches, where only the manual one includes the vesicle membrane. In order to determine the difference between the inner (which corresponds to the diameter that is measured by the macro) and outer diameter altogether 80 random samples were analyzed in various *C*. *elegans* tomograms as shown in Fig 3A and 3B. Fig 3C represents all measured discrepancies as a histogram.

The results show an average discrepancy of 8.9 ± 1.36 nm which is about the expected value of 8 nm, the thickness of two double lipid bilayers. For compensation of the discrepancy of the vesicle membrane and a better comparability of manual and automated measurements the difference of 8.9 nm was added to the results of the automated analysis. The outcome is illustrated as box plots in Fig 2D. Automatically annotated vesicles show a mean of 51.79 ± 5.78 nm for 4dpf and a mean of 50.33 ± 5.88 nm for 8dpf embryonic vesicle diameters which results in a discrepancy of 1.46 nm. Table 1 compares the required time for manual and semi-automated reconstruction of vesicle pools. The required time was estimated from our experience in manual analysis of tomograms as they were used for this work.

**Table 1:** Tabular schedule showing required time for semi-automated reconstruction.

	IMOD	3D ART VeSElecT
**Annotation**	~ 4–8 h **(manual)**	< 7 min **(automated)**
**Proof-reading**	not necessary	~ 0–30 min **(manual)**
**readout of results**	within seconds **(automated)**	within seconds **(automated)**

Table 1 compares the required time for manual segmentation using IMOD (left) and automated segmentation with 3D ART VeSElecT (right) plus the additional manual proof-reading. The required time was measured for each of the 10 here used zebrafish tomograms. The time is split into different working steps of vesicle pool reconstruction, which are: vesicle annotation, proof-reading and readout of results. One typical example for a NMJ with a size of 0.153 μm^3^, with 389 true positive vesicles and 8 false positive vesicles required a runtime of the recognition macro of 6:15 min, 10 min of proof-reading and about 20 seconds for the measurement macro.

As shown in Table 1 the automated vesicle registration with additional manual proof-reading is much less time consuming than entire manual annotation. Manual reconstruction using IMOD needs approximately 4 to 8 hours, depending on the amount of vesicles in one vesicle pool and on the level of experience of the executing person. In contrast, semi-automated vesicle reconstruction needs about 7 to 40 minutes depending on the necessity of manual editing. The manual editing step in our analysis differed from 0 to 30 min, depending on the amount of false positive particles. The following table shows the error rate of each zebrafish tomogram after manual erasure of false positive particles:

Table 2 shows the error rate for each zebrafish tomogram used in our analysis. The error rate was defined as FN / total number of vesicles represented in percent. Additionally, we show error rates as precision and recall. Table 2 shows a very small error rate of 1% to 3.6% (average error is 2.2%) for eight of ten tomograms. Only two tomograms show a much higher error rate of 12.9% and 14.7%. Counting all ten tomograms, the average error rate rises to 4.8%.

**Table 2:** Error rate of zebrafish tomograms.

	Tomo1	Tomo2	Tomo3	Tomo4	Tomo5	Tomo6	Tomo7	Tomo8	Tomo9	Tomo10
Error rate (%)	3.6	1.8	1.0	1.8	2.6	3.3	14.7	12.9	1.2	2.5

Table 2 contains single error rates for every zebrafish tomogram. The error rate is defined as: false negatives (FN) / number of vesicles in the tomogram.

### Application example: *C*. *elegans* septin mutant

After successful proof of principle and rigorous testing 3D ART VeSElecT was next applied on an unresolved biological question. Therefore, we focused on the well described NMJ of *C*. *elegans* and compared wildtype (N2) vesicle pools to alterations in mutants of candidate synaptic vesicle pool regulators–the septins.

**Fig 4:**
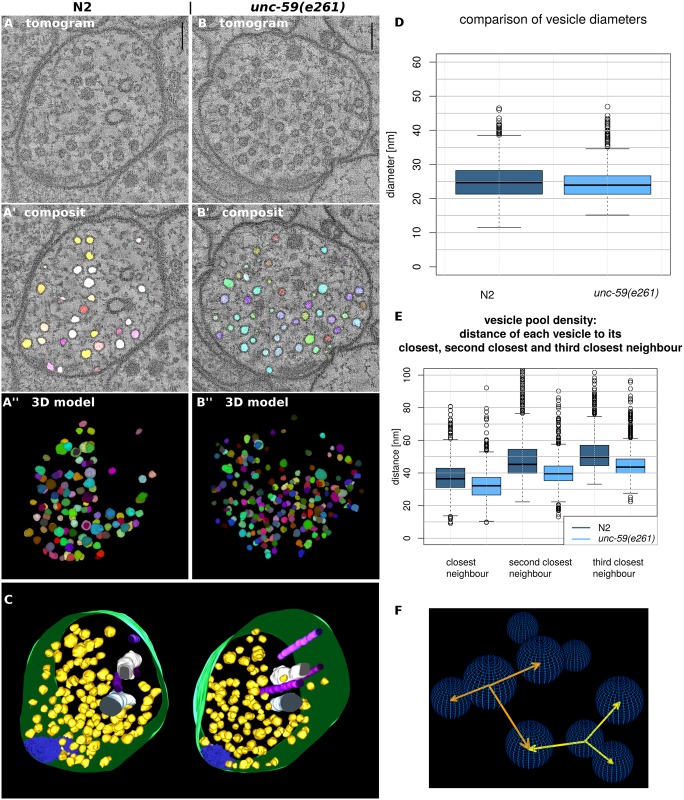
Comparison of N2 and *unc-59(e261) C*. *elegans* tomograms using 3D ART VeSElecT. Fig 4 shows steps of vesicle annotation using 3D ART VeSElecT on N2 (A-A”) and *unc-59(e261)* (B-B”) *C*. *elegans* tomograms. In (A, B) the initial tomogram is illustrated, (A’, B’) show the composite stack that includes the original tomogram plus the annotation of the vesicle pool (vesicles are shown in arbitrary colors). The three dimensional model of the semi-automated annotation (automated registration plus manual proof-reading) of the vesicle pool is shown in (A”, B”). In (C) the semi-automated vesicle pool annotation of (A’) was transferred to IMOD where further structures of the NMJ were complemented. Vesicles are shown in yellow, cell membranes are green, dense projections are blue, endoplasmatic reticulum is white and microtubules are in shades of purple. In (D) synaptic vesicle sizes of N2 and *unc-59(e261)* were compared. Figure (E) illustrates the comparison of vesicle pool density by determining the three closest vesicles (N2 is illustrated in dark blue, *unc-59(e261)* is illustrated in light blue). In (F) the approach for determination of the vesicle pool density is illustrated. It is shown that for each vesicle the distance of its center to the center of its three closest neighbors is calculated. (scale bars = 100 nm).

Because of the so far unsolved cause for the uncoordinated movement in *C*. *elegans* we had reason to believe that through to a loss-of function mutation in the septin gene the ultrastructure of NMJ may be altered. To check whether further laborious research will be profitable and to show that the macros also work on tomograms of various organisms, we used 3D ART VeSElecT as tools to quickly analyze *unc-59(e261)* NMJ for obvious differences of the mutant in comparison to N2 wildtype *C*. *elegans*. Results of this analysis are shown in Fig 4.

Fig 4 shows visual results of the vesicle reconstruction (Fig 4A–4A” and 4B–4B”) as well as the results of the measurements of vesicles sizes and vesicle pool density (Fig 4D and 4E). Fig 4A and 4B show the initial tomogram for N2 as well as *unc-59(e261) C*. *elegans* NMJs. In an automated way the macro finds and marks vesicles based on the tomogram data. Fig 4A’ and 4B’ show composite stacks, i.e., the initial tomogram overlayed with the vesicle annotation as resulted from the macro analysis. Annotation of vesicles can also be shown as three dimensional reconstruction as demonstrated in Fig 4A” and 4B”.

After automated registration and manual improvement (erasure of false positive vesicles) the second macro was used to extract relevant quantitative data like vesicle diameters, vesicle volumes and distances between vesicles. In order to test for differences in the SV pools between *unc-59(e261)* and N2 *C*. *elegans*, average vesicle diameters as well as average vesicle distances to the three closest surrounding vesicles were compared (Fig 4D and 4E). Fig 4D shows the comparison of SV size of N2 and *unc-59(e261)* worms. For the comparison the average diameter calculated out of diameter 1, 2 and 3, which are computed by the second macro, was used. Boxplots in Fig 4D show a small but highly significant (p-value < 0.001) difference in the average SV size of ~0.7 nm between *unc-59(e261)* and N2.

Box plots in Fig 4E show the density of SV pools. Vesicle pool density was determined by measuring the distance of the center of each vesicle to the center of its three closest neighbors, as schematically demonstrated in Fig 4F. As a result, *unc-59(e261)* NMJ show for each measurement (closest, second closest, third closest neighbor) a highly significant (p-value < 0.001) ~4 nm shorter distance between SVs than N2 NMJ.

After using 3D ART VeSElecT the results of the macro can be transformed and transferred to IMOD, where complementation of missing vesicles or other cellular structures is possible (Fig 4C).

### Further analysis of error rates: Precision and recall

**Fig 5:**
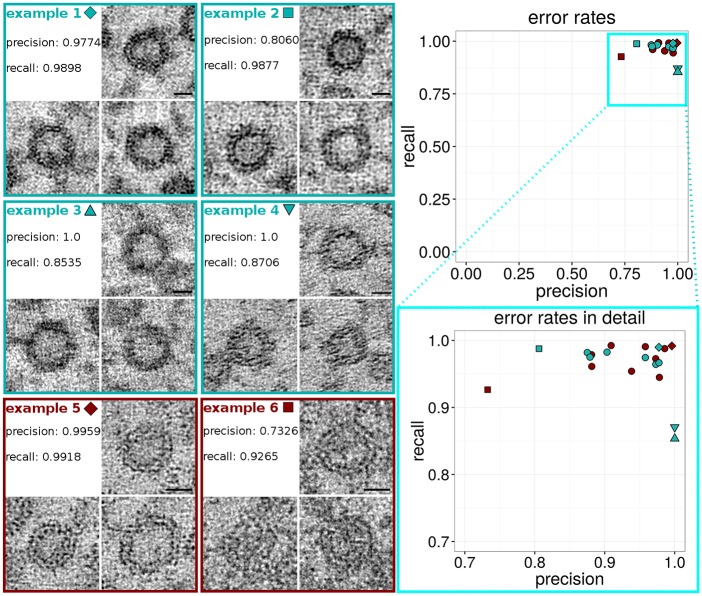
On the right, upper side a dot plot is shown, representing zebrafish (turquoise) as well as *C*. *elegans* (dark red) error rates. Each dot represents precision and recall of one tomogram (magnified detail plot on the lower right side). Tomograms that are shown as examples on the left side of the figure are represented as a special sign (e.g. triangle or square) instead of a round dot in the plot. On the left side, example 1 shows vesicles of a high quality tomogram, with good results for recall (low number of false negatives) and precision (low number of false positives). In comparison to one tomogram with worse precision (example 2) and two tomograms (example 3 and 4) with a higher rate of false negative vesicles, i.e. lower recall. Furthermore, examples 5 and 6 show vesicles of one tomogram with best precision and recall rate (example 5) in comparison to one tomogram with very low precision. Scale bars: 20 nm.

To better understand and determine differences in tomogram quality, as well as the resulting error rates, that strongly depend on the quality of the tomograms, we show and discuss vesicles of various tomograms in Fig 5 as well as error rates for each tomogram as precision and recall.

On the right side of Fig 5 the results for precision and recall are plotted. Each dot represents precision and recall for one tomogram. All dots cluster in the right upper corner (close to x = 1 and y = 1) of the dot plot. This indicates that error rates of the macro are low, hence 3D ART VeSElecT provides reliable results, with only few inaccuracies. Error rates for *C*. *elegans* are within the same range as for zebrafish. Outliers were compared to tomograms with best results for precision and recall on the left side of the figure.

Precision and recall are two parameters expressing error rates. Precision is an indicator for the number of false positives (FP) in relation to true positives (TP) (Precision = TP / (TP + FP)). Whereas recall indicates the number of false negatives (FN) in relation to TP (Recall = TP / (TP+FN). A precision and recall rate of 1 would implicate an accuracy of the macros performance of 100%. Often samples have either a very good result for recall or for precision. Example 1 has both a very good rate for precision as well as recall (precision = 0.9774, recall = 0.9898). The tomogram extracts of vesicles (Fig 5, example 1), show the very good quality of the tomogram. The texture of the tomogram is very smooth and the double lipid membrane of the vesicles can be easily recognized. Example 2 has also a very good result for recall (0.9877) but lacks precision (0.8060). Comparing the pictures, example 1 shows a smoother texture than example 2. This seems to be the reason for the different recognition of false positive particles. Examples 3 and 4 have both very good results for precision (precision = 1, no false positives), however they show lower rates for recall (example 3: recall = 0.8535 and example 4: recall = 0.8706). Example 3 obviously lacks quality, compared to example 1. Details like the double lipid membrane of the vesicles cannot be recognized. The vesicle membranes appear blurry. Because of the undefined edges, some vesicles are not recognized in this tomogram. Example 4 has only a slightly better result for recall than example 3, but at first sight the vesicles of the tomogram look more defined than in example 3. When comparing example 4 to the examples 1 and 2, notable differences in the texture of the tomograms are obvious. Whereas the texture in example 1 and 2 appear like small round dots, the texture of example 4 looks like small lines, which are oriented in one direction. There are several possibilities that influence tomogram quality. First, small aberrations of the sample and small technical anomalies, such as vibrations, during the tilt-series acquisition can influence the quality of the resulting tomogram. Second, ideal settings regarding exposure time and focus are crucial. Third, imperfections in the tomogram processing, like inexact overlay of the single-tilts for creating a double-tilted tomogram due to uneven distribution of fiducials, are crucial for the quality of the final tomogram and may be explanations for this observation.

Example 5 shows vesicles of the *C*. *elegans* tomogram with the best results for precision and recall (precision = 0.9959, recall = 0.9918). Although contrast is much lower than compared to examples 1 and 2, the double lipid layer can be recognized very well. In contrast example 6 represents diverse vesicles of a tomogram with low precision (many false negative particles). The vesicle in the upper right corner of example 6 was recognized by the macro, whereas both vesicles underneath have not been recognized. These two vesicles are hardly distinguishable from the background, because of the low contrast of vesicle center and membranes. Therefore they are even hard to recognize for the human observer. In this case we decided to include the above discussed, lower quality tomograms in the analysis, because besides of a certain error rate, vesicle recognition was still within our tolerance. Tomograms always have variations in quality, because of the many factors that have an influence on the result, on the one hand, during tilt-series recording, and on the other hand, at the tomogram processing step. Hence, the user himself has to set a threshold and decide whether a tomogram is still suitable for his analyses, therefore precision and recall are helpful indications.

## Discussion

Fiji macro 3D ART VeSElecT was developed to study synaptic vesicles in electron tomograms. It successfully quantifies vesicle properties (i) in embryonic *Danio rerio* 4 and 8 days past fertilization (dpf) and (ii) compares *Caenorhabditis elegans* N2 wild-type NMJ with septin (*unc-59(e261))* mutants. The tool allows detecting and quantifying development-specific and mutant-specific changes in synaptic vesicle pools in both models.

There is fully automated vesicle registration and characterization implemented in the Fiji macros. The division of the workflow into two macros (one for registration and one for characterization) allows in particular reducing false positives by an optional manual revision step in between the two macros. Also the RoiManager plugin collects fully automatically results in colour and has the possibility of manual removal of false-positive vesicles. Our tool was evaluated regarding performance (detected vesicles) and specificity (true vesicles) as well as precision and recall with small error rates. We also demonstrate gain in segmentation and morphological filtering compared to *ilastik* (see supplement, S1 Fig). Moreover, there is a large time gain compared to manual segmentation with IMOD. The speed gain of our tool can be up to 68 times faster in comparison to manual annotation.

Our results demonstrate that established differences in vesicle sizes, which have been analyzed manually in *D*. *rerio* embryos [22], could be confirmed by the macro. Manual and semi-automated annotation of the same vesicle pool looked similar. We proved that the macro is a very useful tool for fast automatic recognition and annotation of vesicles of NMJ. Notably, original vesicle sizes of the automated annotation are smaller than the manual results (see Results Figs 2D and 3). The differences appear because the methods have divergent approaches for measurement and can be explained by four reasons. First, for manual annotation of vesicles, the outer membrane served as boundary for the vesicle size, whereas the automated annotation of the macros includes only the inner vesicle volume minus the vesicle membrane. To compensate this difference we added the ascertained value of the difference of inner and outer vesicle diameter. After that, results of the automated annotation are slightly bigger but about the size as in the manual results. Furthermore, the manual approach is subjective and depending on the judgments of the executing person and therefore is prone to interpretational mistakes. For that reason manually annotated datasets should not be directly compared to automatically analyzed datasets. Third, another explanation for discrepancies in the results is that errors may occur in the macros’ recognition step, like over- or underestimation of the vesicle size (see below, advantages and limitations of the algorithm). Finally, underestimation of automated segmentation tools is a known phenomenon, which is also mentioned in other publications and can be in part explained by the fact that automatic segmentation is more “accurate” [40].

Nevertheless we were able to confirm the discovery of smaller vesicle sizes in 8dpf zebrafish embryos compared to 4dpf embryos by applying 3D ART VeSElecT. Although the detected differences are smaller than for the manual measurements, the results are still highly significant (p-value < 0.001).

In a second experiment we demonstrated further possibilities of applications of the macros. Therefore we compare datasets of septin *unc-59(e261)* loss-of-function mutants to N2 wildtype *C*. *elegans*. The analysis revealed differences in vesicle sizes. Synaptic vesicle diameters of N2 NMJ are in average ~0.7 nm bigger than in *unc-59(e261)*. Furthermore vesicle pools in *unc-59(e261) C*. *elegans* show a higher density. For each neighboring vesicle (first, second and third) a ~4 nm bigger distance in N2 NMJ was detected compared to *unc-59(e261)*. The loss-of-function mutation seemed to have an effect of the architecture of the vesicle pool which hints to a role of septin UNC-59 in the organization of the vesicle pool.

It is known, that UNC-59 and UNC-61 bind each other and build heterodimers thus assembling to higher order structures [41]. Despite their ability of assembling, septins are able to bind to membranes and have been shown to co-purify with synaptic vesicles [42]. Our hypothesis is that septins may play a role in the formation of the filament structures interconnecting the vesicle pool, which could explain the obtained differences in vesicle pool density. The underlying molecular reasons for the morphological alterations of the septin mutant are not the focus of this work, but rather serve as an example for the application of the algorithm.

### 3D ART VeSElecT: Advantages and limitations

In comparison to other published automated tools that were designed for connectomics, e.g., for synapse detection [43] or detection of vesicle clusters by use of Radon-like features [44], 3D ART VeSElecT is specially designed to find, annotate, characterize and quantify single vesicles in vesicle pools of high resolution electron tomograms (here: 20 000 x– 40 000 x magnification). As an advantage 3D ART VeSElecT is implemented as user friendly tool which can easily be used by people that are not familiar with programming. The software is created as two coherent connected macros for the open source software Fiji with defined parameters that can be adjusted depending on vesicle size and sphericity. This approach has both advantages and disadvantages. First, because it is not trainable software, it saves the labor of time consuming training steps. While the specificity of the macro to recognize round, vesicle-like structures in electron-tomograms restricts the software’s application possibilities to this task, it is necessary for the reliable recognition of the vesicles, since the recognition of structures in electron tomograms is a challenging task even for humans and even more for computers because of the low signal-to-noise ratio. An alternative possibility would have been to detect vesicles by template matching with spherical structures of different size, as previously applied for filament-like structures [12], however such an algorithm requires a large number of convolution operations over the entire raw non-binarized volume, which would be much less efficient compared to our approach, where most operations are performed on the binary volume after applying a threshold. The software accomplishes the automatic recognition and reconstruction of vesicles almost perfectly with very small average error rates (Fig 5) of unrecognized particles and particles with obvious over- or underestimation of the vesicle size (see Table 2). For comparison, hand- annotation gains 100% of vesicles but takes up to 68 times longer and is prone to mistakes when determining vesicle sizes. The number or characteristic of the error using 3D ART VeSElecT strongly depend on the quality of the electron tomogram as presented in Table 2. If the tomogram lacks quality, the software’s error rate rises. This is why we used only high-quality double tilted tomograms for the analysis. In case of the analysis of zebrafish tomograms, the two tomograms with error rate of 12.9% and 14.7% were included. Considering, that few missing vesicles are not influencing the extensive analysis results in this case, where vesicle size was in focus, and all detected vesicles were as well recognized as in tomograms with lower error rate.

Besides that, sometimes the macro falsely recognizes other cellular structures as “synaptic vesicles”. Such false positive particles can be removed manually with the “erase” function in the RoiManager, and therefore are excluded from final statistical results, because of their irrelevance for the final error rate. Furthermore, in very few vesicles, sizes are over- or underestimated. Overestimation can happen if vesicles lie very close to each other, or if conspicuous filament structures are surrounding the vesicle. Underestimation can particularly happen in vesicles of insufficient quality or with no perfectly clear core. Sometimes very few vesicles are recognized as two halves. In this case both particles can be manually merged in the RoiManager.

After application of the recognition macro, a second macro is used which creates a new 3D model (“StackSegmented” / “StackSegementedCopy”) and “Composit”-stack which include the manual improvements. In addition the second macro extracts data like vesicle size as diameter 1, 2 and 3 (diameters in x, y and z orientation; ordered after size), feret (biggest diameter) as well as the volume of each vesicle and density of the vesicle pool by measuring the distance of each vesicle to its x closest neighbors (x is variable). The results shown in the StackSegmented can be converted to a model file and afterwards be opened in IMOD. This way, missing particles as well as other cellular structures can be manually added to the 3D model if necessary.

### Further considerations and development

The macros can be applied to check various questions concerning synaptic vesicle pools, but may also be a useful tool for other round structures in electron tomograms, like lipid droplets or Golgi apparatus vesicles. For these alternative structures again specific fine-tuning of the program is necessary for optimal performance. Furthermore we demonstrated that the macro works on tomograms of synapses of various organisms and is therefore broadly applicable for synaptic analysis. Another advantage is the potential transfer and use of our automated reconstruction in IMOD for further manual completion of the 3D model. Fiji and IMOD are both freely available software, therefore the application of the macro is possible for everybody and not linked to any expense. As described above, manual segmentation is prone to mistakes but also automated recognition has a certain error rate. This is the reason why an automated recognition followed by a manual review step, as in our presented workflow, seems to be the best way to efficiently create accurate, reproducible, nonbiased data.

The macro works on one Fiji version on Linux which we provide on this web page www.bioinfo.biozentrum.uni-wuerzburg.de/computing/3DART-VeSElecT together with a user description and a simple tutorial.

Next we want to further extend and enhance the performance and application possibilities of the macro, e.g., for recognition and differentiation of other organelles and architectural components in the cell. Another demanding example currently tested are tomograms of alpha granules and other vesicles in platelets. An implementation of radon-like features [42] for enhancement of pattern recognition would be an interesting possibility to further improve the macros performance.

## Conclusion

In summary, we present possibilities and advantages of the here developed automated, fast performing analysis tool “3D ART VeSElecT”, which is specifically recognizing round 3D particles in electron tomograms, with a speed gain of up to 68 times compared to the manual approach (via IMOD). The macro generates independent, nonbiased and reproducible results. Therefore, double-blind analyses become unnecessary. Optional additional manual filtering is available. A gain compared to *ilastik* is in segmentation and manual filtering. The macros can be used for extensive, so far very time consuming or not within reach analysis of electron tomograms or for fast prescreening of datasets, e.g., in developmental questions or cellular neurobiology. Therefore, it solves the bottle-neck problem of the time consuming reconstruction step. 3D ART VeSElecT can perform approximately up to 68 times faster than manual analysis while producing very few errors.

## Materials and Methods

### Requirements for application of 3D ART VeSElecT

Fiji version: 1.51g
Additional plugin: 3D ImageJ Suit [37]

Analysis was performed on a PC with the following characteristics:

Processor: Intel^®^ Core^™^ i5-3570 CPU @ 3.4GHz
Working storage: 16 GB
System type: 64 bit operating system

Both macros the “3DART_VeSElecT_RegistVesicle” as well as the “3DART_VeSElecT_MeasureVesicle” work on the latest Fiji version 1.51g.

### 3D ART VeSElecT

The code of the macro is freely available for download at www.bioinfo.biozentrum.uni-wuerzburg.de/computing/3DART-VeSElecT.

Automated vesicle registration and characterization was implemented in Fiji 1.51g as two macros (registration and measurement) to reduce false positives by a manual editing step in between. Preprocessing and contrast enhancement work on image-stacks of 1nm/pixel in x and y direction. Semi-automated cell selection was integrated. 3D ART VeSElecT removes interfering components, detects vesicles by 3D segmentation, calculates vesicle volume and diameter (spherical approximation, inner/outer diameter), results are collected using the RoiManager plugin including automatic removal of non-matching confounder vesicles.

### Used parameters for vesicle exclusion

There are three user-defined constraints on the detected vesicles (volume, sphericity, elongation) that are set at the beginning of the macro. They determine how many detected objects pass the filter and are recognized as vesicles in the end, and thus can be used to tune the error rate. Using sloppy constraints will increase recall but decrease precision.

#### Zebrafish tomogram analysis (further details in [22]).

Min. volume (V) < 6500 (30k tomograms) or 5000 (20k tomograms)
Min. sphericity (S) < 0.5
Maximum ratio between major and second radius of a fitted ellipse (max. elongation) > 2.0

#### *C*. *elegans* tomogram analysis

Min. volume (V) < 3000
Min. sphericity (S) < 0.5
Maximum ratio between major and second radius of a fitted ellipse (max. elongation) > 2.0

### Animals

*C*. *elegans* wild-type (Bristol *N2*) as well as *unc-59(e261)* septin mutants were used and maintained using standard methods [32]. For the experiments only young adult worms were used.

### High pressure freezing (HPF), freeze substitution (FS) and embedding

HPF and FS for morphological analysis were conducted as previously described for *C*. *elegans* [3], [45]. For HPF the Leica EM HPM100 was used. Up to 10 *C*. *elegans* young adult worms were put in one freeze chamber (Leica Microsystems GmbH, Wetzlar, Germany; specimen carriers type A, 100 μm, covered with specimen type B, 0 μm) covered in antifreeze spacer solution of OP50 *E*. *coli* bacteria in M9 minimum medium with 20% BSA. Samples were frozen at >20.000 K/s and >2100 bar which is crucial for the optimal vitrification of the samples. High-pressure frozen pellets were kept in liquid nitrogen and transferred into an EM AFS2 (Leica Microsystems GmbH, Wetzlar, Germany) for freeze substitution. FS was performed as described previously [8] unless epon was used for infiltration and embedding which was performed as flat embedding, where pellets and epon where put in between two ACLAR sheets or other plastic sheets with comparable characteristics.

#### Sectioning and EM preparation

Epon blocks were cut with the Leica EM UC7 (Leica Microsystems GmbH, Wetzlar, Germany) and a histo diamond knife (Diatome, Biel, Switzerland) into 250 nm thick sections for tomography. Slices were transferred on pioloform coated slotted copper grids (Plano GmbH, Wetzlar, Germany).

Afterwards grids were stained and contrasted in 2.5% uranyl acetate in ethanol for 15 min and Reynolds [46] lead citrate for 10 min. All grids were coated with an approximately 5 nm thin carbon layer using a MED 010 (Balzers Union AG, Balzers, Liechtenstein). For double-tilt series gold fiducials were required for accurate overlay of the series. Therefore copper grids were incubated for 15 min in an undiluted solution 12 nm ProtA-Au-beads (Dianova GmbH, Hamburg, Germany) followed by a single washing step in distilled water. Samples were checked for proper morphological tissue preservation and screened for ice-crystal formation. Ice-crystal formation can be first detected in nuclei where ice nucleation mostly starts. In case of detected ice-crystal formation, samples were omitted from further analysis.

#### EM and electron tomography

For EM tomography a JEM-2100 (JEOL, Munich, Germany) transmission electron microscope equipped with a TemCam F416 4k x 4k (Tietz Video and Imaging Processing Systems, Gauting, Germany) was used. For automated analysis double tilts were necessary because of their better resolution. Therefore two tilt series from at least +65° to -65° in 1° increment steps were recorded from the same NMJ using SerialEM [47]. Whereas the second tilt series was taken after the rotation of the grid by 90°. The tilt-series were aligned, combined and reconstructed using weighted back projection algorithms of the eTomo software [10], which is part of the IMOD software package [11].

#### Statistical analysis

For all statistical analysis of significance, Mann-Whitney-Wilcoxon test in R [48] was used. Results are highly significant if p-value < 0.001.

## Supporting Information

S1 Supplement The supplement includes further data, downloads (tutorial, user description and software) and comparison of 3D ART VeSElecT to *ilastik*. (DOCX)

S1 Fig (a) Slice through the original tomogram at z = 55, (b) same slice after preprocessing and thresholding by our method, (c) vesicle membrane probability maps produced by *ilastik*, (d) same probability maps after applying Otsu’s algorithm for thresholding. (e) Segmented vesicles at z = 55, and (f) max-intensity projection of entire volume after segmentation using our method, (g) max-intensity projection of segmented volume using *ilastik*-generated probability maps as input, and (h) max intensity projection of segmented volume after applying 3D watershed directly to the probability maps. Scale bar = 100 nm.
(TIFF)
