# Supplementary material for: FIJI Macro 3D ART VeSElecT: 3D Automated Reconstruction Tool for Vesicle Structures of Electron Tomograms
Source: PLoS Comput Biol. 2017 Jan 5;13(1):e1005317. doi: 10.1371/journal.pcbi.1005317 (PMC5289597; doi:10.1371/journal.pcbi.1005317)
Supplement: S1 Supplement — (DOCX) [file pcbi.1005317.s001.docx]

**S1 Supplement:**

All requirements, needed to test 3D ART VeSElecT are freely available at our homepage: www.bioinfo.biozentrum.uni-wuerzburg.de/computing/3DART-VeSElecT. Here we provide as downloads: both macros “3DART_VeSElecT_RegistVesicle.ijm” and “3DART_VeSElecT_MeasureVesicle.ijm” as one zip file, a user description, a test stack and a Fiji version 1.51g for Linux operating systems.

**Comparison to *ilastik* as an example for learning based methods**

To better assess the performance of our method in comparison to other tools, we trained a random forest classifier in *ilastik*. We selected features targeting round, blob-like structures (*Gaussian Smoothing, Laplacian of Gaussian, Difference of Gaussians,* and *Hessian of Gaussian Eigenvalues*) with large sigma (*σ=3.5…10.0 px*). Training data were labeled manually in 3D in a subvolume of 686 x 674 x 111 pixels.


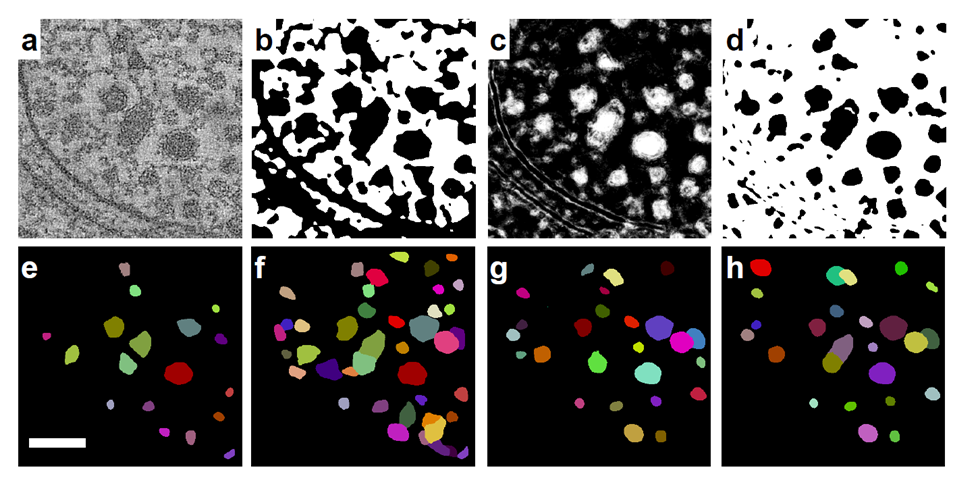


**S1 Fig: (a) Slice through the original tomogram at z=55, (b) same slice after preprocessing and thresholding by our method, (c) vesicle membrane probability maps produced by *ilastik*, (d) same probability maps after applying Otsu’s algorithm for thresholding. (e) Segmented vesicles at z=55, and (f) max-intensity projection of entire volume after segmentation using our method, (g) max-intensity projection of segmented volume using *ilastik*-generated probability maps as input, and (h) max intensity projection of segmented volume after applying 3D watershed directly to the probability maps. Scale bar = 100 nm.**

The mask produced by thresholding the *ilastik*-generated vesicle probability maps (d) shows higher specificity for vesicles compared to the mask produced by our method (b). After running the segmentation and morphological filtering part on both masks, more vesicles are found by our method (f, 43 vesicles) compared to using the *ilastik*-generated mask (g, 26 vesicles) or applying the 3D watershed directly on the mask (h, 26 vesicles). Vesicles recognized by all three methods look identical in size and shape. The fact that fewer vesicles are recognized using the probability map, and that different vesicles but the same overall number are found depending on the approach (g, h) shows that the learning-based method is not superior to our targeted method. Since false positives are easier to correct for than false negatives, a less specific classification would be preferable (and could probably be obtained using different parameters and training data). While the time needed for sparse labeling of training data is negligible, the runtime of the classifier on the small 3D test dataset used here still exceeds that of the macro by a factor of at least 5 (tested on an Intel i7-2640 CPU @2.80GHz with 8GB RAM).

In summary, the learning-based approach shows comparable performance when used as replacement to the preprocessing and filtering steps in our pipeline, but cannot replace the segmentation and morphological filtering part. The probability maps still require morphological filtering as performed by our segmentation pipeline. This is probably because the non-vesicle structures have identical contrast and texture in the tomograms. Training an additional object-level classifier would be necessary for a fully learning-based analysis pipeline. In the case of vesicles however, where clear morphological criteria can be defined a priori, this does not present the most efficient approach.

This may be different for scenarios where less well-defined features need to be segmented. Recent improvements in learning-based methods such as convolutional networks generally outperform targeted methods in terms of precision and recall at the cost of high demands on computation time and training data. We did not perform a comparison to deep-learning based approaches, since the scope of our work was to develop an integrated, user friendly and freely distributed solution for segmentation and quantification of vesicles, including a manual proofreading step. This enables researchers without detailed knowledge in image processing to analyze vesicle pools in 3D EM data.

**S1 Table: comparison of 3D ART VeSElect to *ilastik***

|  | **Fiji / 3D ART VeSElecT** | ***ilastik*** |
| --- | --- | --- |
| **Required user input** | 3 parameters* | Labeled training data |
| **output** | Pixel level segmentation and results table | Probability map |
| **runtime** | 5 min | 35 min (plus labeling) |

*the 3 parameters are: min. volume, min. sphericity and max. elongation
