# Supplementary figures and images for: FIJI Macro 3D ART VeSElecT: 3D Automated Reconstruction Tool for Vesicle Structures of Electron Tomograms
Source: PLoS Comput Biol. 2017 Jan 5;13(1):e1005317. doi: 10.1371/journal.pcbi.1005317 (PMC5289597; doi:10.1371/journal.pcbi.1005317)

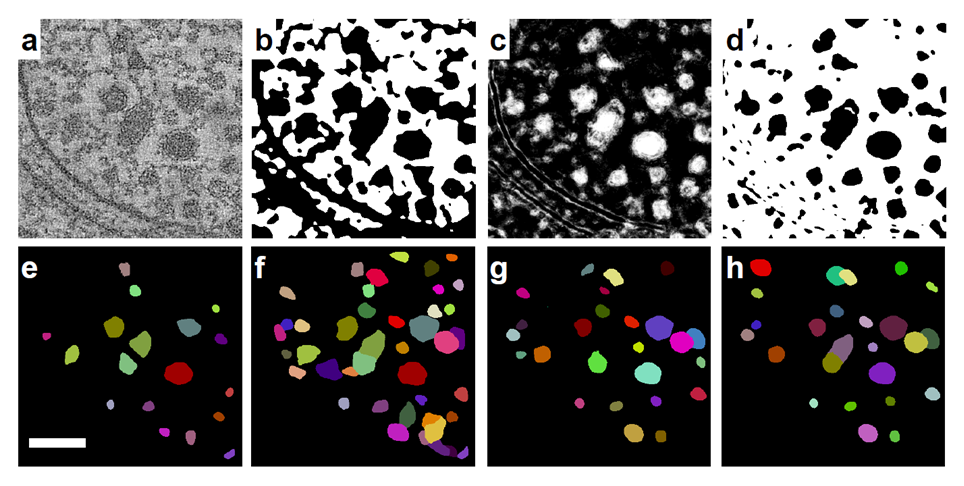

Supplement: S1 Fig — (a) Slice through the original tomogram at z = 55, (b) same slice after preprocessing and thresholding by our method, (c) vesicle membrane probability maps produced by ilastik, (d) same probability maps after applying Otsu’s algorithm for thresholding. (e) Segmented vesicles at z = 55, and (f) max-intensity projection of entire volume after segmentation using our method, (g) max-intensity projection of segmented volume using ilastik-generated probability maps as input, and (h) max intensity projection of segmented volume after applying 3D watershed directly to the probability maps. Scale bar = 100 nm. (TIFF) [file pcbi.1005317.s002.tiff]
